# Supplementary material for: Alternatively spliced STIM2.3 is an evolutionarily late store-operated Ca2+ entry regulator expressed in brain
Source: J Cell Sci. 2026 Apr 21;139(8):jcs264353. doi: 10.1242/jcs.264353 (PMC13143212; doi:10.1242/jcs.264353)
Supplement: Supplementary information [file joces-139-264353-s1.pdf]

Table S1. Expression vectors

Construct	Fluorescent marker	Vector
**HA-STIM2.2**	-	pEX-SP1
**HA-STIM2.3**	-	pEX-SP1
**mKate2-STIM2.2**	mKate2, N-term	pEX-SP1
**mKate2-STIM2.3**	mKate2, N-term	pEX-SP1
**mCherry-STIM1**	mCherry, N-term	pEX-SP1
**STIM2.2-mCherry**	mCherry, C-term	pEX-SP1
**YFP-STIM2.2**	YFP, N-term	pEX-SP1
**YFP-STIM2.3**	YFP, N-term	pEX-SP1
**YFP-STIM2.2Δ5K**	YFP, N-term	pEX-SP1
**YFP-STIM2.2Δ674**	YFP, N-term	pEX-SP1
**YFP-STIM2.2 2xIP**	YFP, N-term	pEX-SP1
**YFP-STIM2.2 2xIP+Δ5K**	YFP, N-term	pEX-SP1
**YFP-STIM2.2Δ711**	YFP, N-term	pEX-SP1
**YFP-STIM2.2Δ675-710**	YFP, N-term	pEX-SP1
**NFAT1-GFP**	GFP	pCAGGS
**HA-STIM2.2-YFPc**	HA, N-term; YFPc, C-term	pEX-SP1
**STIM2.2-YFPn**	YFPn, C-term	pBabe
**HA-STIM2.3-YFPc**	HA, N-term; YFPc, C-term	pEX-SP1
**STIM2.3-YFPn**	YFPn, C-term	pBabe
**STIM1-YFPn**	YFPn, C-term	pBabe

Journal of Cell Science • Supplementary information

**Orai1-YFPn**	YFPn, C-term	pMax
**Orai2-YFPn**	YFPn, C-term	pMax
**Orai3-YFPn**	YFPn, C-term	pMax
**EFEMP1-YFPn**	YFPn, C-term	pBabe
**pAAV-mgreenLantern**	GFP	Addgene<br>#164469

Journal of Cell Science • Supplementary information

Table S2. Primer pairs for qRTPCR

Target	Primer forward	Primer reverse
hSTIM1	Hs_STIM1_1_SG / QT00083538	Qiagen QuantiTect Assay
hSTIM2	Hs_STIM2_1_SG / QT00023744	Qiagen QuantiTect Assay
hSTIM2.3	GAGCTCAGCTTGCTCCACAC	CGAGCCCAAGGTGAATACAT
hSTIM2.2	GAGCTCAGCTTGCTCCACAC	TGGCACTTCCCATTGCTTTTC
Orai1	ATGAGCCTCAACGAGCACT	GTGGGTAGTCGTGGTCAG
Orai2	TGGAACTGGTCACCTCTAAC	GGGTACTGGTACTGCGTCT
Orai3	GTACCGGGAGTTCGTGCA	GGTACTCGTGGTCACTCT
TBP	Hs_TBP_1_SG / QT00000721	Qiagen QuantiTect Assay
RNApol	Hs_POLR2A_1_SG / QT00033264	Qiagen QuantiTect Assay

Table S3. Applied primary and secondary antibodies

Antigen	Manufacturer	Catalog #	clonality	Dilution
**α- phospho-AMPK**	Cell signaling	2535	polyclonal	1:1000
**α-AMPK**	Cell signaling	2532S	polyclonal	1:1000
**α-β-Actin**	Sigma Aldrich	A5441	monoclonal	1:5000
**α-HA 3F10**	Roche	11867423001		1:1000
**α-STIM1**	Proteintech	11565-1-AP	polyclonal	1:1000
**α-STIM2**	Alomone	ACC-064	polyclonal	1:300
**FluoTag®-X4 α-GFP**	NanoTag Bio.	N0304-At488-L	SdAbs	1:500
**α-tRFP**	Evrogen	AB233	Rabbit	1:500
**α-Synapsin**	SynapticSystems	106 004	Guinea pig	1:500
**α-PSD95**	DSHB	K28/43	Monoclonal	1:250
**α-Neurofilament L**	SynapticSystems	171014	Guinea pig	1:500
**α-MAP2**	SynapticSystems	188004	Guinea pig	1:500
**α-rabbit-HRP**	GE Healthcare/Amersham	NA9340		1:10000
**α-rat-HRP**	Sigma Aldrich	A5795		1:20000
**α-mouse-HRP**	Amersham/Bioscience	NA931		1:5000
**α-rabbit AF568**	Invitrogen/Thermo Fisher	A-11011		1:500

Table S4. ENCODE accession ID's and supplementary information for Fig. S1.

Accession ID	Experiment ID	File Type	BioSample	Organism
**ENCFF269DXG**	ENCSR317LMH	.fa	muscle of arm tissue female embryo (98 days)	H. sapiens
**ENCFF629SLX**	ENCSR406YML	.fa	muscle of arm tissue male embryo (91 days)	H. sapiens
**ENCFF442QQD**	ENCSR620ZNQ	.fa	muscle of arm tissue female embryo (120 days)	H. sapiens
**ENCFF851VPL**	ENCSR806ESH	.fa	muscle of back tissue female embryo (98 days)	H. sapiens
**ENCFF326BCF**	ENCSR729ZII	.fa	muscle of back tissue male embryo (91 days)	H. sapiens
**ENCFF093TMQ**	ENCSR995ORR	.fa	muscle of back tissue female embryo (105 days)	H. sapiens
**ENCFF038OLY**	ENCSR096USV	.fa	muscle of leg tissue male embryo (127 days)	H. sapiens
**ENCFF591YBG**	ENCSR860DST	.fa	muscle of leg tissue male embryo (96 days)	H. sapiens
**ENCFF969AEA**	ENCSR545WAC	.fa	muscle of leg tissue male embryo (97 days)	H. sapiens
**ENCFF098SSN**	ENCSR265NZF	.fa	spleen tissue embryo (112 days)	H. sapiens
**ENCFF696UCL**	ENCSR700QVJ	.fa	spleen tissue male embryo (120 days)	H. sapiens

RNA Seq data was downloaded from ENCODE for skeletal muscle (arm, leg and back) and spleen (Table S4). Isoform-level quantification was performed using Salmon (1) and kallisto (2). The reference transcriptome was obtained from Gencode (hg38 v29). The log2-transformed, mean transcripts expression levels were visualized as heatmaps. rMATS (3) was used to perform exon-usage analysis. Sashimi plots were used to display RNA Seq read densities at exon and junction regions.

References:

1. Patro, Rob, et al. "Salmon provides fast and bias-aware quantification of transcript expression." Nature methods 14.4 (2017): 417-419.

2. Bray, Nicolas L., et al. "Near-optimal probabilistic RNA-seq quantification." Nature biotechnology 34.5 (2016): 525-527.

3. Shen, Shihao, et al. "rMATS: robust and flexible detection of differential alternative splicing from replicate RNA-Seq data." Proceedings of the national academy of sciences 111.51 (2014): E5593-E5601.

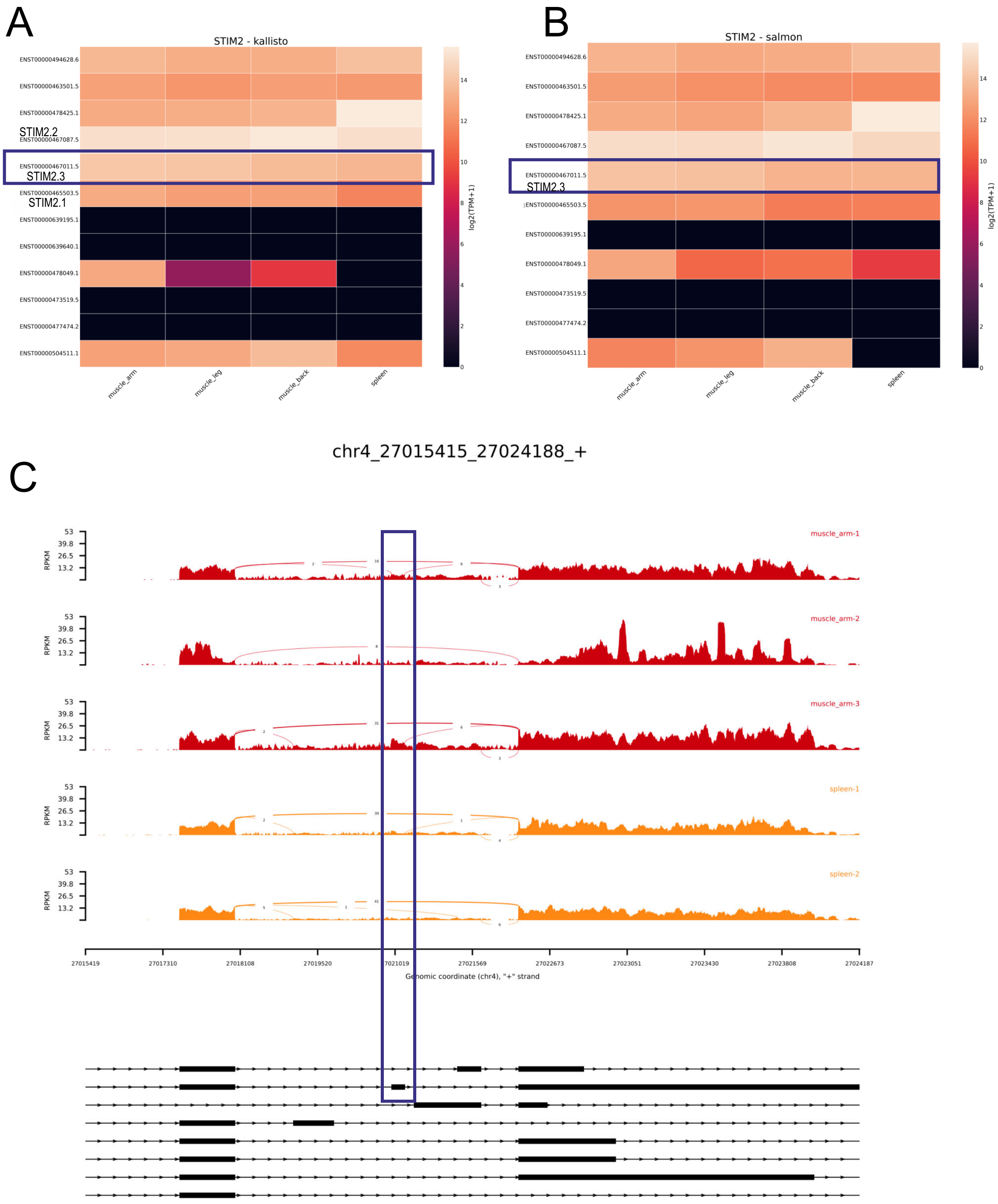

Fig. S1. Isoform and junction-level analyses of human muscle and spleen

A. Isoform level expression analyses using software package Kallisto; RPKM (Reads Per Kilobase Million)
B. Isoform level expression analyses using software package Salmon; RPKM (Reads Per Kilobase Million)
C. Junction level quantification using rRMATS. Blue box denotes location of STIM2.3 encoding exon.

Journal of Cell Science • Supplementary information

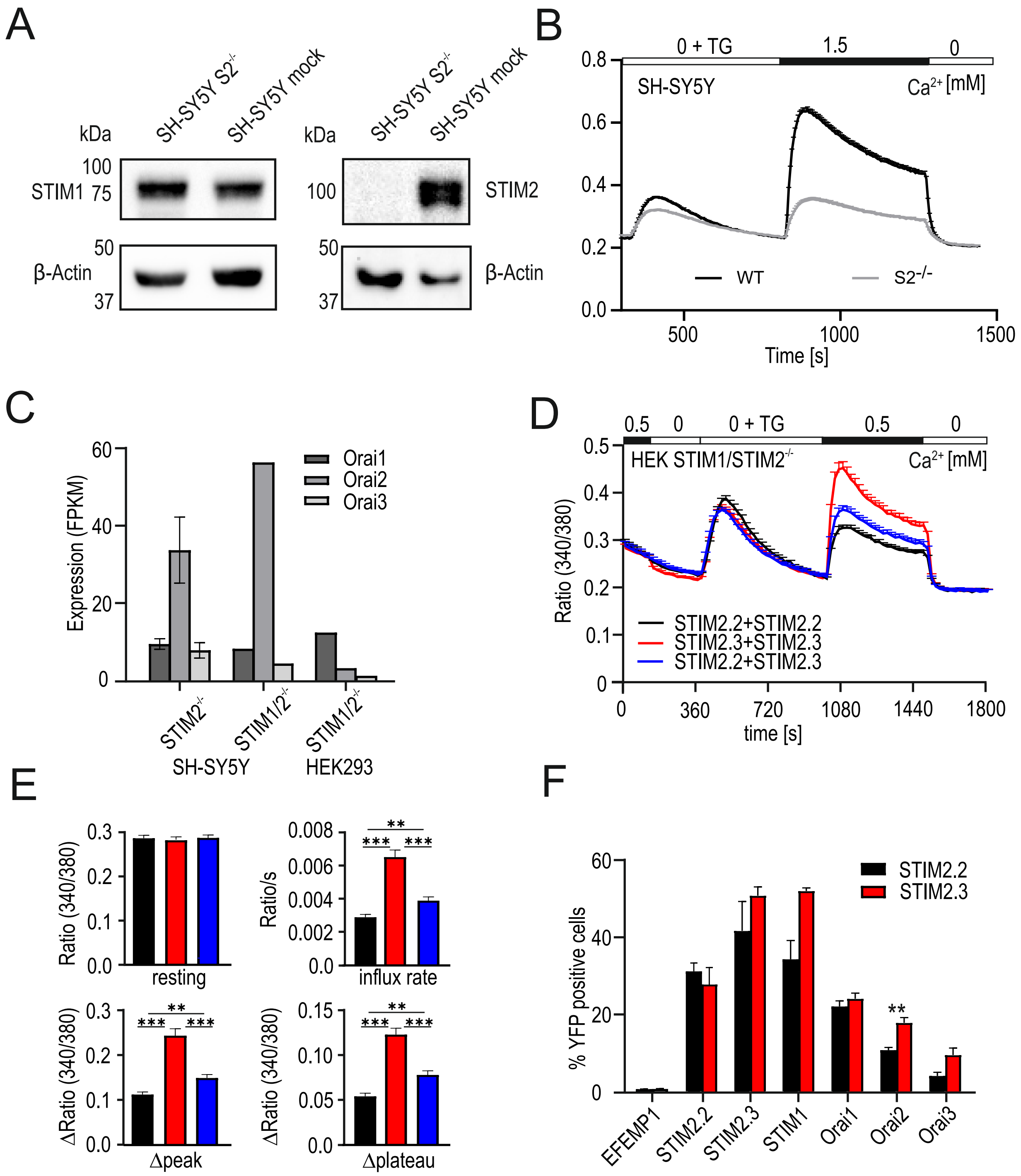

Fig. S2. Generation of STIM2 -/- SHSY-5Y cells, Expression and Co-expression analyses

A. Western blot demonstrating absence of STIM2 in SH-SY5Y STIM2$^{-/-}$.

B. Average traces showing changes (mean+SEM) in intracellular Ca^{2+} (Ratio 340/380) over time in response to perfusion of different external Ca^{2+} [mM] as indicated in the upper bar of SH-SY5Y Wt and STIM2$^{-/-}$ cells.

C. FPKM (Fragments Per Kilobase of transcript per Million mapped fragments) values of Orai 1-3 genes in SH-SY5Y STIM2$^{-/-}$, SH-SY5Y STIM1/2$^{-/-}$ and HEK STIM1/2$^{-/-}$ cells from RNA Seq analysis.

D. Average traces showing changes (mean+SEM) in intracellular Ca^{2+} (Ratio 340/380) over time in response to perfusion of different external Ca^{2+} [mM] as indicated in the upper bar in HEK STIM1/2$^{-/-}$ cells transfected with YFP-STIM2.2+mKate-STIM2.2 (black, n=100), YFP-STIM2.3+mKate-STIM2.3 (red, n=77) and YFP-STIM2.2+mKate-STIM2.3 (blue, n=99).

E. Quantification of changes in resting Ca^{2+}, influx rate, peak and plateau measured in C. *** $p< 0.001$, ** $p< 0.01$; Kruskal-Wallis ANOVA.

F. Interaction of STIM2.2-YFPc (black) or STIM2.3-YFPc (red) with POI-YFPn in HEK STIM1/2$^{-/-}$ cells was quantified as % YFP positive cells with bimolecular fluorescence complementation via flow cytometry. Data (mean+SEM) was obtained from 3 independent transfections with 10,000 measured cells each.

Journal of Cell Science • Supplementary information

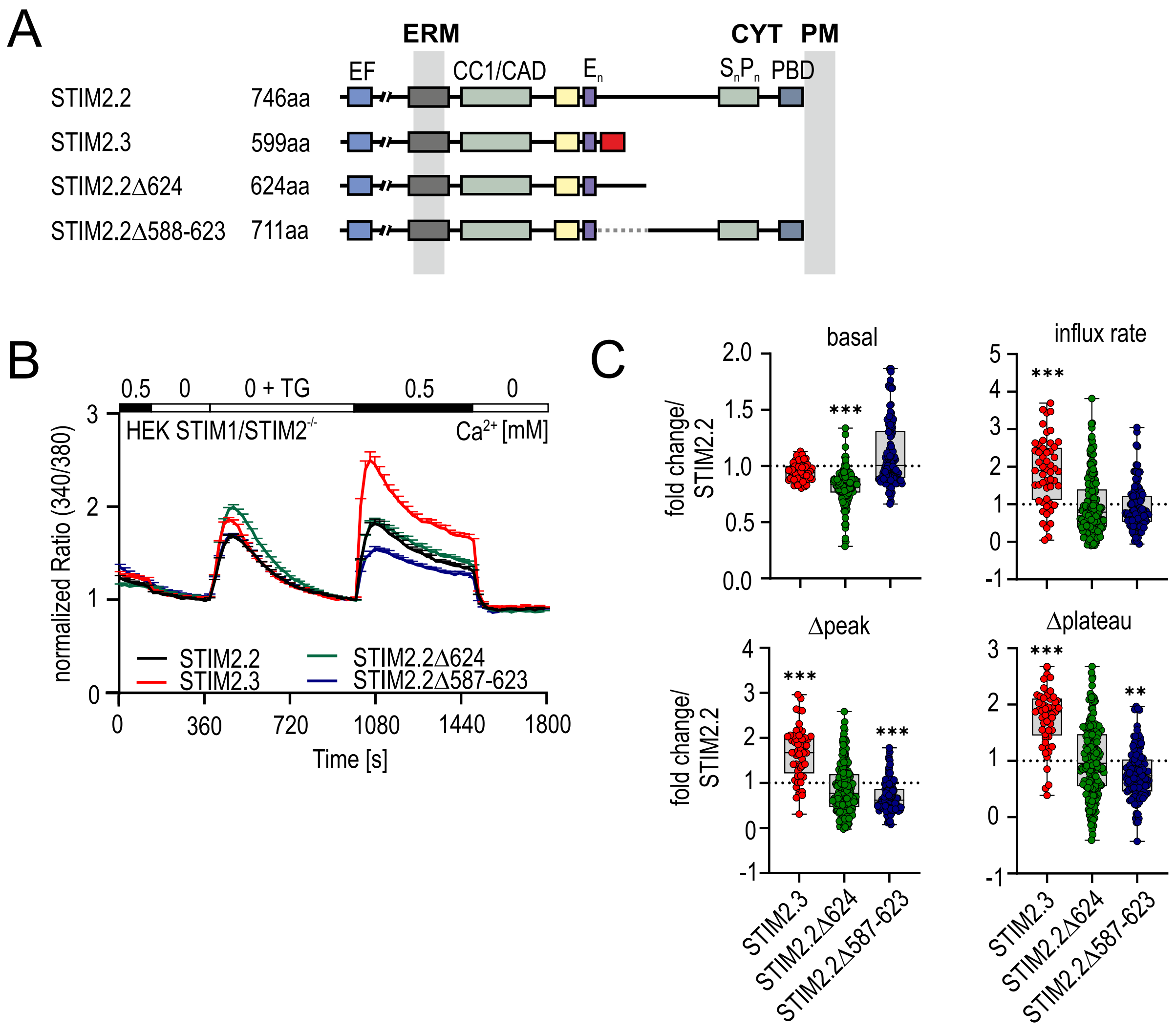

Fig. S3. Additonal structure-function analyses of STIM2 C-terminal domains

A. Schematic protein structure with functional domains of STIM2.2, STIM2.3, STIM2.2Δ624 terminating after aa624 and STIM2.2Δ588-623 with an internal deletion of 35 aa.

B. Normalized average traces of intracellular Ca^{2+} (Ratio 340/380) over time in response to perfusion with different external Ca^{2+} [mM] as indicated in the upper bar after transfection with YFP-STIM2.2 (black, n=165), YFP-STIM2.3 (red, n=207), YFP- STIM2.2Δ624 (green, n=187) or YFP-STIM2.2Δ588-623 (blue, n=117) in HEK STIM1/2$^{-/-}$ cells.

C. Quantification of changes in resting Ca^{2+}, influx rate, Δ peak and Δ plateau measured in B as fold change normalized to STIM2.2. *** p< 0.001, ** p< 0.01; Kruskal-Wallis ANOVA.

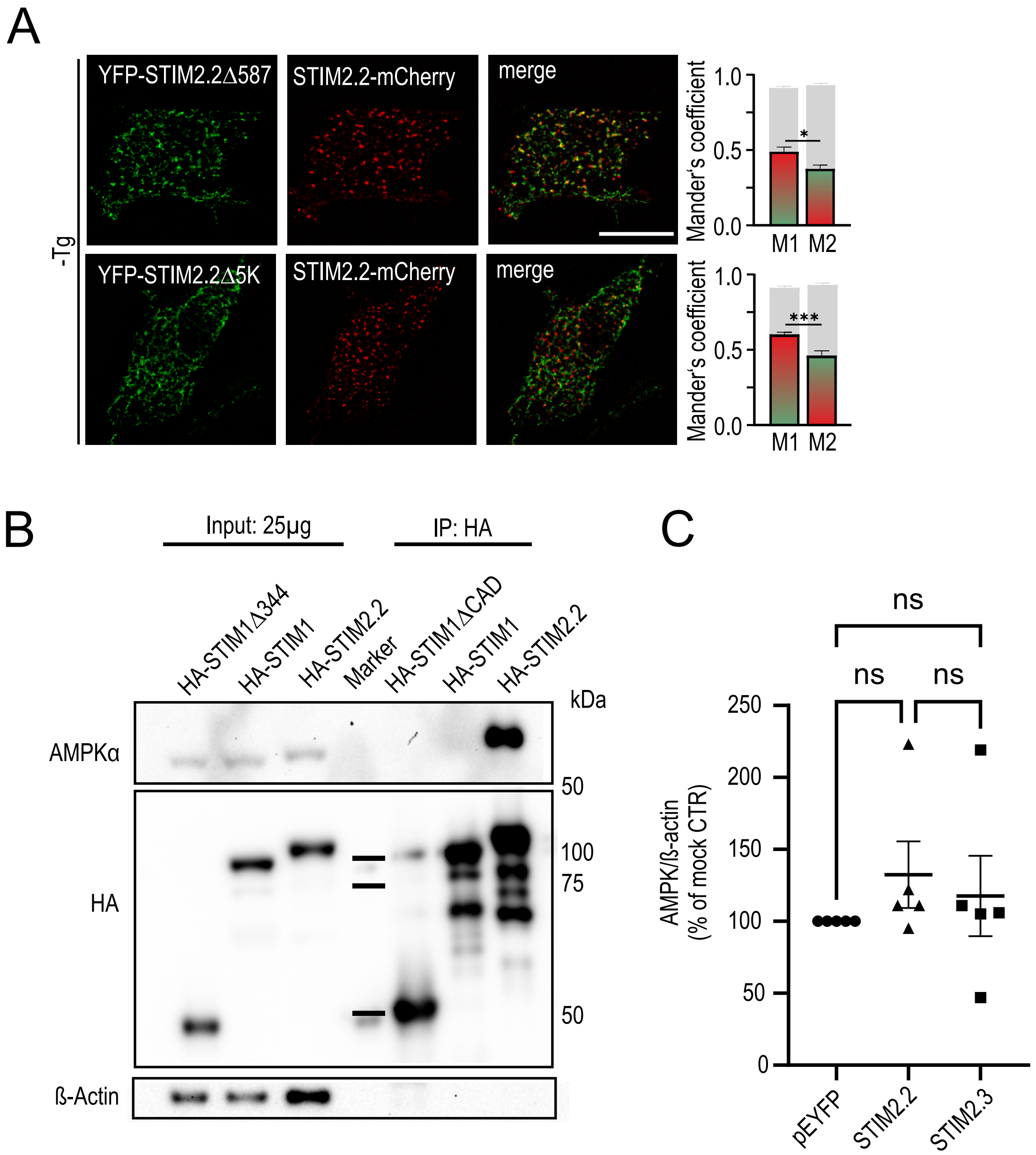

Fig. S4. Colocalization analyses of STIM2.2 with additional deletion constructs and Co-immunoprecipitation controls

A. Representative images of HEK STIM1/2$^{-/-}$ cells co-expressing STIM2.2-mCherry (red) and YFP-STIM2.2Δ587 (green) (upper panel) or STIM2.2 Δ5K (green) (lower panel) and merged images before Thapsigargin (-Tg). Scale bar indicates 10µM. For each condition 16-29 cells from 3 independent transfections were analyzed using Mander's overlapping coefficients (M1, M2): STIM2.2+ Δ587 M1: 0.48 M2: 0.37; STIM2.2+ Δ5K M1: 0.58 M2: 0.45. Mander's overlapping coefficients of YFP-STIM2.2 and STIM2.2-mCherry transfected cells are indicated by grey bars, M1: 0.90 M2: 0.92. * $p < 0.05$; *** $p < 0.001$, Mann-Whitney test.

B. Immunoprecipitation of HA-STIM1Δ344, HA-STIM1 or HA-STIM2.2 transfected cells with endogenous AMPKα in HEK STIM1/2$^{-/-}$ cells using anti-HA agarose. Membrane was incubated with the indicated antibodies and developed sequentially.

C. Quantification of AMPKα input normalized to ß-Actin signal of transfected cells analysed in Fig. 8.

Journal of Cell Science • Supplementary information

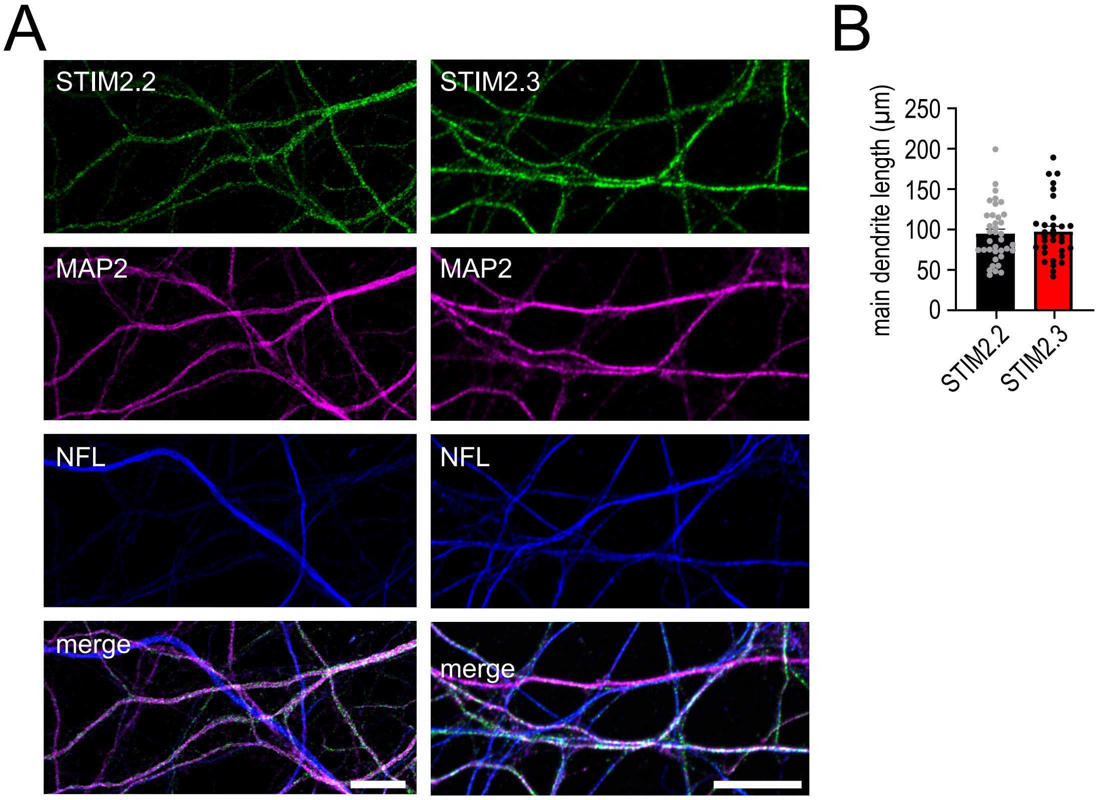

Fig. S5. Morphological features of STIM2.x expressing hippocampal neurons

A. Representative images of primary neuronal cultures expressing mkate-STIM2.2 or mkate-STIM2.3 and stained with anti-RFP (green), anti-MAP2 and anti-NFL (blue). Scale bar indicates 10 µm.

B. Quantification of mean dendrite lengths of STIM2.2 or STIM2.3 expressing neurons.

Journal of Cell Science • Supplementary information

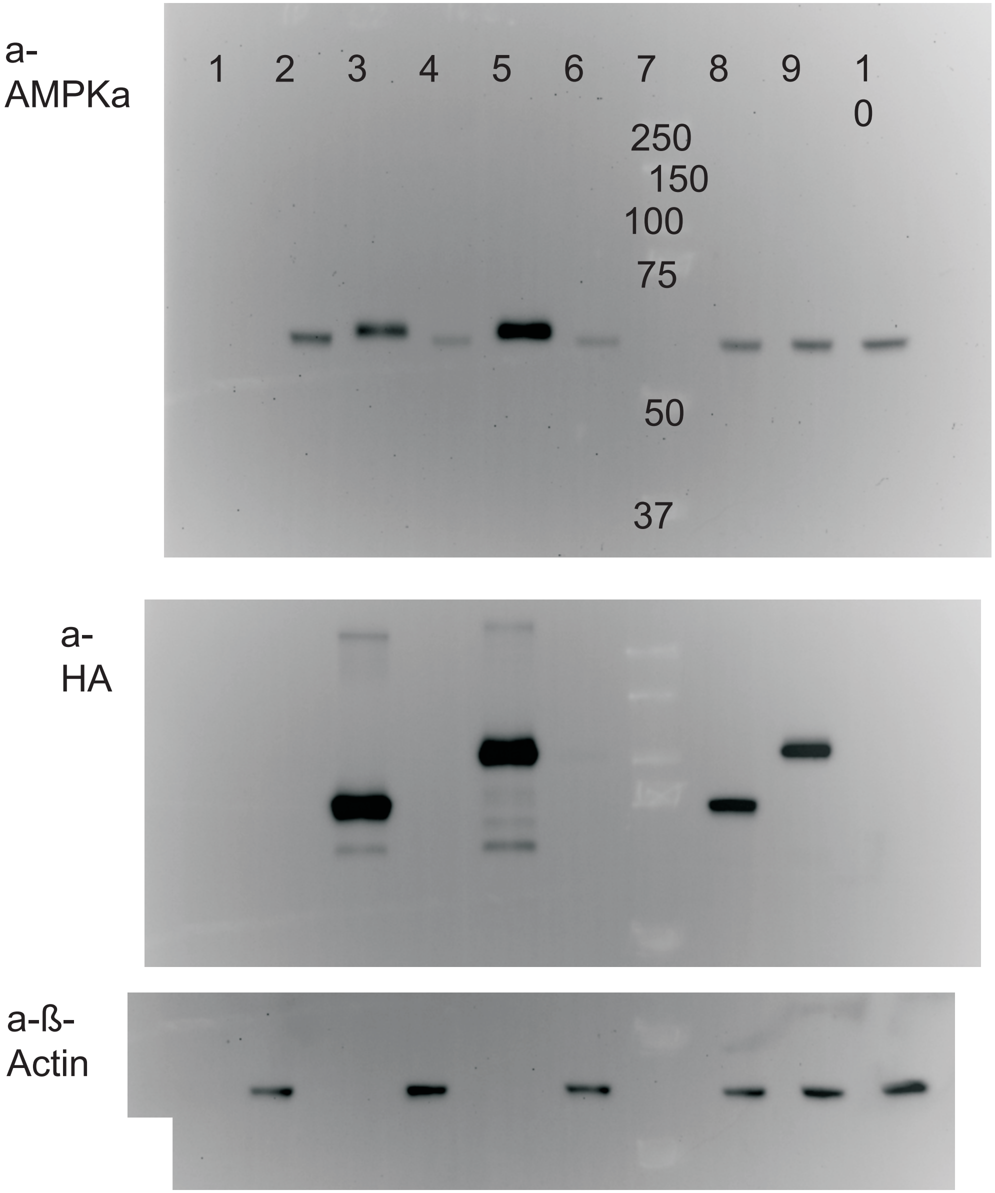

WB number	284
Development date	17.03.2022
Experiment title:	Co-IP HEK DKO +S2 endogen AMPK

Experiment:
Transfection HEK DKO JetOptimus, 4µg DNA STIM2/pEYFPC1
lysis in DM IP buffer 30min 4°C end-over-end rotating; amount protein/IP= 700-1000µg, 25µl HA-agarose, 4°C over night end-over-end rotating; next day washing: 4x 5min in 500µl wash buffer RT, centrifugation 500xg 30s; elution 25µl Tris-HCl pH 6,8+2%SDS (freshly prepared) 5min 90°C shaking
Input and FT 25µg protein
8,5% gel, PVDF

Loading scheme of gel A
1. E pEYFPC1
2. FT pEYFPC1
3. E STIM2.3
4. FT STIM2.3
5. E STIM2.2
6. FT STIM2.2
7. dual color
8. STIM2.3
9. STIM2.2
10. pEYFPC1

Primary Antibody: AMPKa,Cell signaling, 1:1000; HA3F10, Roche, 1:1000; ß-Actin, 1:5000 (Gene, company, dilution)

Secondary antibody: rabbit, 1:10000; rat 1:10000; mouse 1:5000 (Species, dilution)

Journal of Cell Science • Supplementary information

WB number	290
Development date	01.04./04.04./05.04.2022
Experiment title:	Co-IP STIM2+endo AMPK

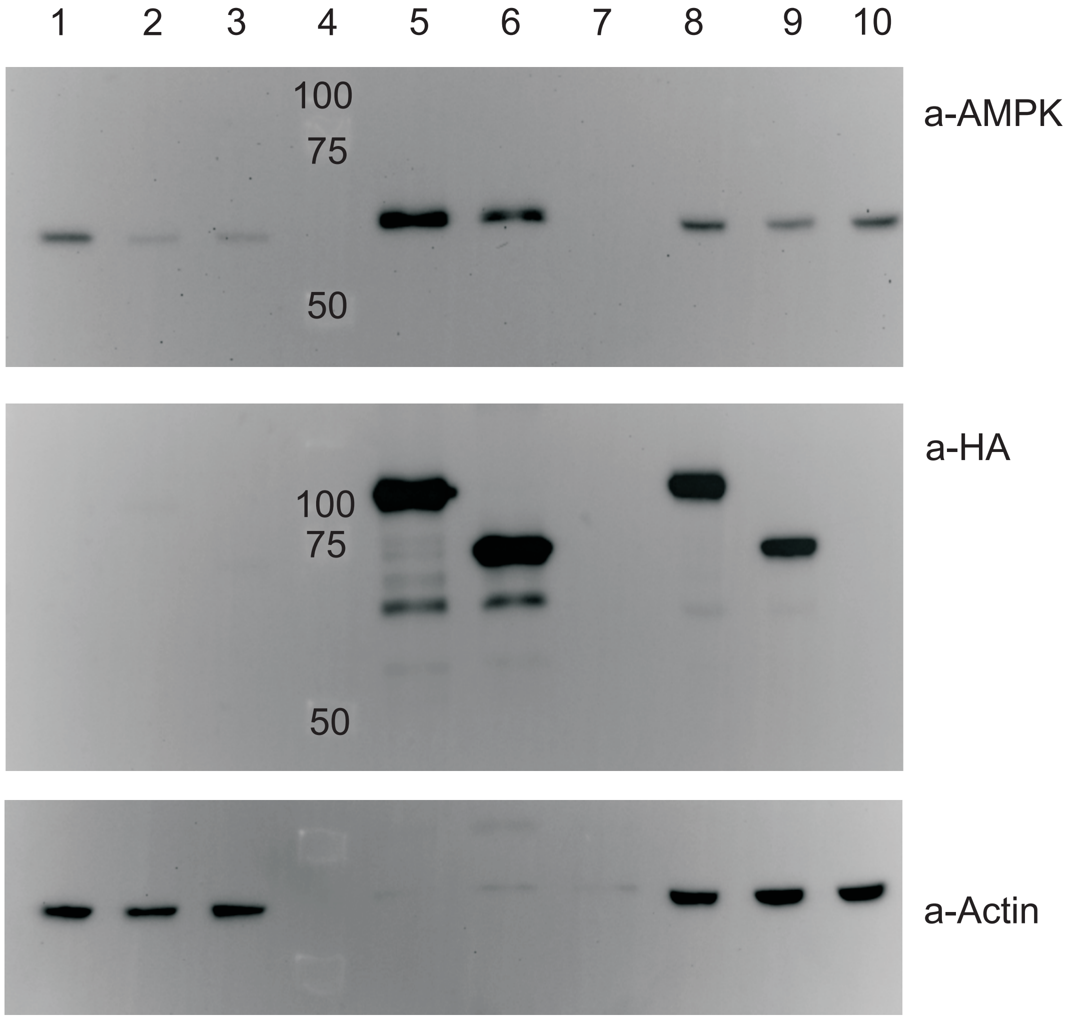

Experiment:
Transfection HEK DKO Jet Optimus 3µg DNA /6cm dish
Lysis: 30min 4°C rotating
IP: 30µL agarose, 1mg protein, over night 4°C rotating
washing 4x5min 500µL wash buffer RT, 500xg 30s
elution 25µl 50mM Tris-HCl pH 6,8 +2%SDS 5min 90°C shaking
8,5% gel, PVDF; FT and Input 25µg, eluate complete

Loading scheme of gel A
1. FT ctrl
2. FT S2.2
3. FT S2.3
4. dual colorl
5. E S2.2
6. E S2.3
7. E ctrl
8. input S2.2
9. input S2.3
10. input ctrl

Primary Antibody: AMPK, Cell Signaling, 1:1000; HA 3F10, Roche,1:1000; Actin, 1:5000 (Gene, company, dilution)

Secondary antibody :rb, 1:10000; rat, 1:10000; m, 1:5000 (Species, dilution)

WB number	303
Development date	14.04./19.04./20.04.2022
Experiment title:	Co-IP STIM2+endo AMPK (3)

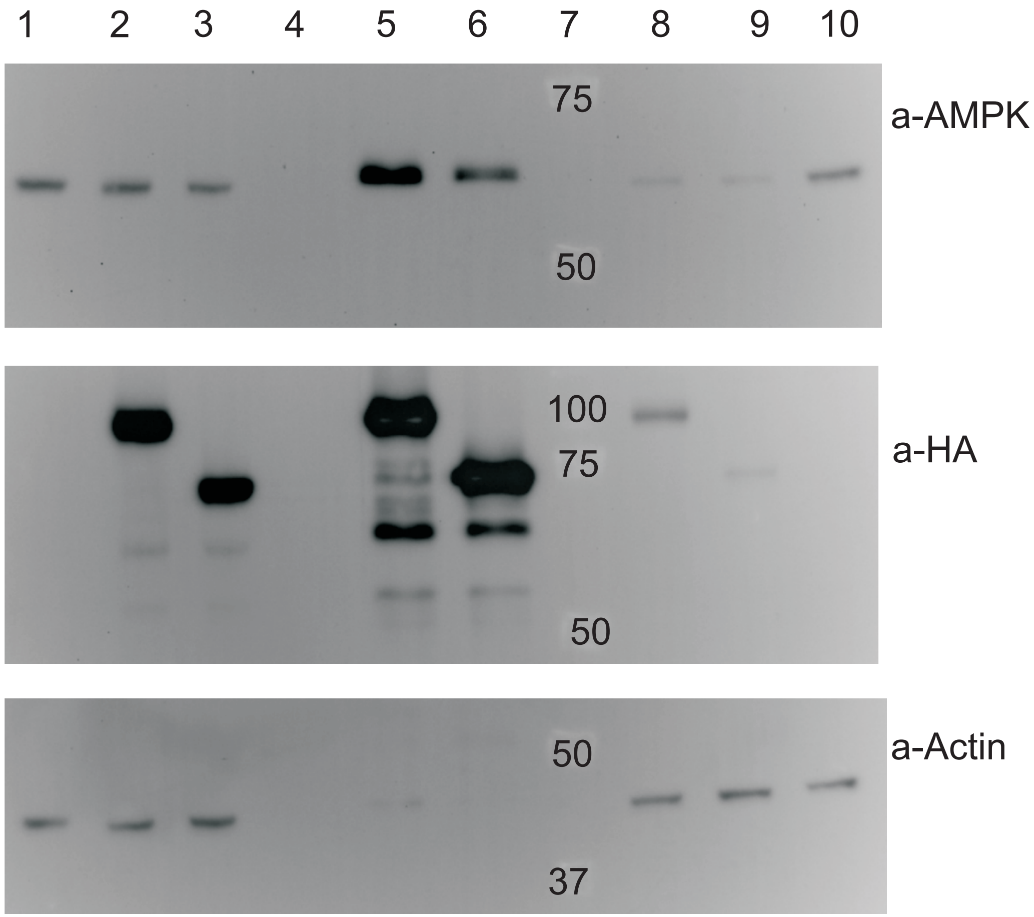

Experiment:
Transfection HEK DKO Jet Optimus 3µg DNA /6cm dish
Lysis: 30min 4°C rotating
IP: 25µL agarose, 1mg protein, over night 4°C rotating
washing 4x5min 500µL wash buffer RT, 500xg 30s
elution 25µl 50mM Tris-HCl pH 6,8 +2%SDS 5min 90°C shaking
8,5% gel, PVDF; FT and Input 25µg, entire eluate

Loading scheme of gel A
1. Input ctrl
2. Input S2.2
3. Input S2.3
4. E ctrl
5. E S2.2
6. E S2.3
7. Dual color
8. FT S2.2
9. FT S2.3
10. FT ctrl

Primary Antibody: AMPK, Cell Signaling, 1:1000; HA 3F10, Roche,1:1000; Actin, 1:5000 (Gene, company, dilution)

Secondary antibody :rb, 1:10000; rat, 1:10000; m, 1:5000 (Species, dilution)

WB number	304
Development date	21.04./22.04./25.04.2022
Experiment title:	Co-IP STIM2+endo AMPK +/-TG

gel A: -TG

1 2 3 4 5 6 7 8 9 10

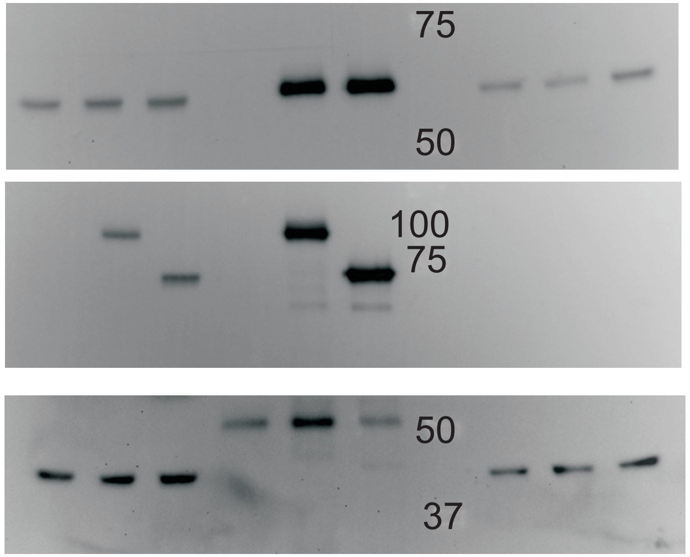

a-AMPK

a-HA

a-Actin

gel B: +TG

1 2 3 4 5 6 7 8 9 10

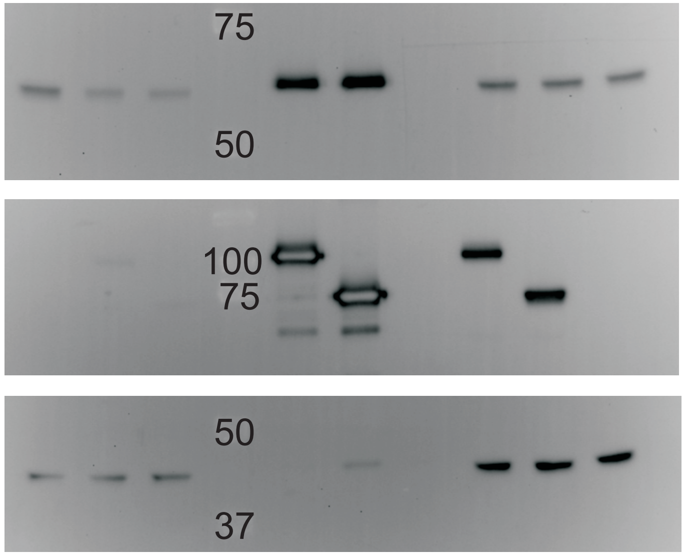

a-AMPK

a-HA

a-Actin

--> probably Tg was not working

Experiment:
Transfection HEK DKO Jet Optimus 3µg DNA /6cm dish
Lysis: 30min 4°C rotating
IP: 25µL agarose, 1mg protein, over night 4°C rotating
washing 4x5min 500µL wash buffer RT, 500xg 30s
elution 25µl 50mM Tris-HCl pH 6,8 +2%SDS 5min 90°C shaking
8,5% gel, PVDF; FT and Input 25µg, entire eluate

Loading scheme of gel A -TG
1. Input ctrl
2. Input S2.2
3. Input S2.3
4. E ctrl
5. E S2.2
6. E S2.3
7. Dual color
8. FT S2.2
9. FT S2.3
10. FT ctrl

Loading scheme of gel B +TG
1. FT ctrl
2. FT S2.2
3. FT S2.3
4. DC
5. E S2.2
6. E S2.3
7. E ctrl
8. Input S2.2
9. Input S2.3
10. Input ctrl

Primary Antibody: AMPK, Cell Signaling, 1:1000; HA 3F10, Roche,1:1000; Actin, 1:5000 (Gene, company, dilution)

Secondary antibody :rb, 1:10000; rat, 1:20000; m, 1:5000 (Species, dilution)

Journal of Cell Science • Supplementary information

WB number	305
Development date	28.04./02.05.2022
Experiment title:	Co-IP STIM2+endo AMPK +/-TG (2)

gel A: +TG

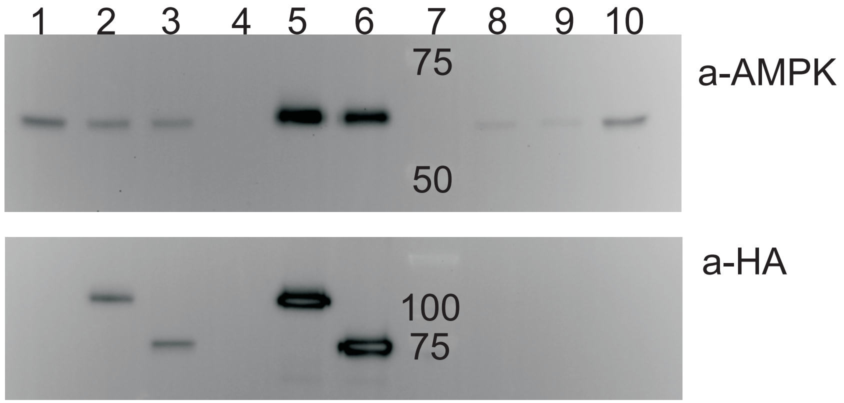

gel B: -TG

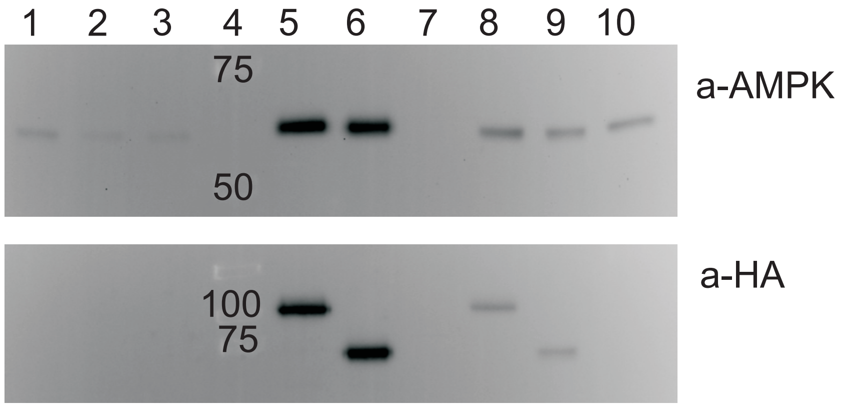

Experiment:
Transfection HEK DKO Jet Optimus 3µg DNA /6cm dish
Lysis: 30min 4°C rotating
IP: 30µL agarose, 1mg protein, over night 4°C rotating
washing 4x5min 500µL wash buffer RT, 500xg 30s
elution 30µl 50mM Tris-HCl pH 6,8 +2%SDS 5min 90°C shaking
8,5% gel, PVDF; FT and Input 25µg, entire eluate

Loading scheme of gel A +TG
1. Input ctrl
2. Input S2.2
3. Input S2.3
4. E ctrl
5. E S2.2
6. E S2.3
7. Dual color
8. FT S2.2
9. FT S2.3
10. FT ctrl

Loading scheme of gel B -TG
1. FT ctrl
2. FT S2.2
3. FT S2.3
4. DC
5. E S2.2
6. E S2.3
7. E ctrl
8. Input S2.2
9. Input S2.3
10. Input ctrl

Primary Antibody: AMPK, Cell Signaling, 1:1000; HA 3F10, Roche,1:1000 (Gene, company, dilution)

Secondary antibody :rb, 1:10000; rat, 1:20000 (Species, dilution)

Journal of Cell Science • Supplementary information

WB number	306
Development date	06.05.2022
Experiment title:	Co-IP AMPK Blot 284/290 stripped

gel A: Blot 284

1 2 3 4 5 6 7 8 9 10

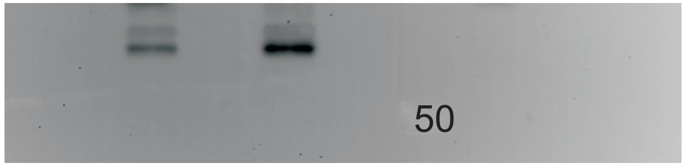

a-Phos-AMPK

Experiment:

cold stripped 20min

Loading scheme of gel A Blot 284
1. E ctrl
2. Input ctrl
3. E S2.3
4. Input S2.3
5. E S2.2
6. Input S2.3
7. Dual color
8. FT S2.2
9. FT S2.3
10. FT ctrl

gel B: Blot 290

1 2 3 4 5 6 7 8 9 10

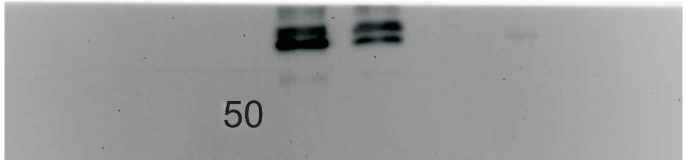

a-Phos-AMPK

Loading scheme of gel B Blot 290
1. FT ctrl
2. FT S2.2
3. FT S2.3
4. DC
5. E S2.2
6. E S2.3
7. E ctrl
8. Input S2.2
9. Input S2.3
10. Input ctrl

Primary Antibody: Phos-AMPK, Cell Signaling, 1:1000 (Gene, company, dilution)

Secondary antibody :rb, 1:10000 (Species, dilution)

Journal of Cell Science • Supplementary information

WB number	314
Development date	24.05.2022
Experiment title:	Co-IP AMPK Blot 303/305-TG stripped

gel A: 303

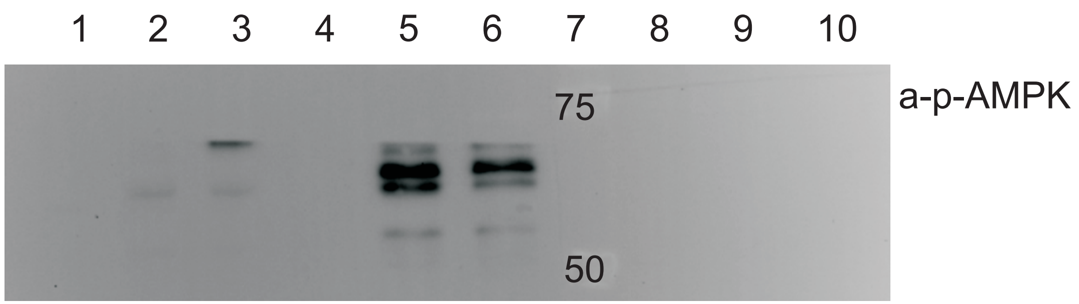

gel B: 305-TG

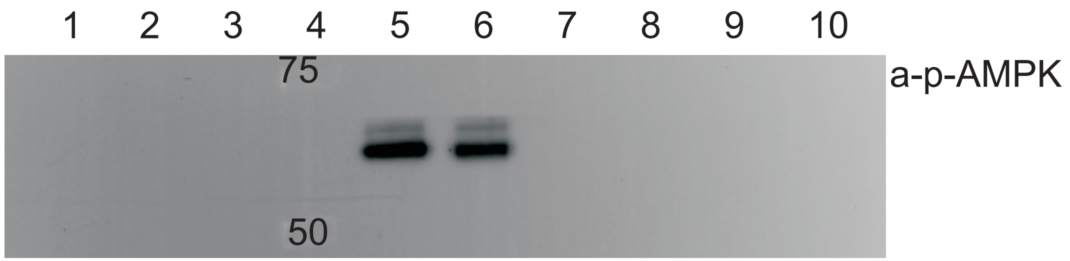

Experiment:
Transfection HEK DKO Jet Optimus 3µg DNA /6cm dish
Lysis: 30min 4°C rotating
IP: 25µL agarose, 1mg protein, over night 4°C rotating
washing 4x5min 500µL wash buffer RT, 500xg 30s
elution 25µl 50mM Tris-HCl pH 6,8 +2%SDS 5min 90°C shaking
8.5% gel, PVDF; FT and Input 25µg, entire eluate

cold stripping 20min

Loading scheme of gel A 303
1. Input ctrl
2. Input S2.2
3. Input S2.3
4. E ctrl
5. E S2.2
6. E S2.3
7. Dual color
8. FT S2.2
9. FT S2.3
10. FT ctrl

Loading scheme of gel B 305 -TG
1. FT ctrl
2. FT S2.2
3. FT S2.3
4. dual color
5. E S2.2
6. E S2.3
7. E ctrl
8. I S2.2
9. I S2.3
10. I ctrl

Primary Antibody: p-AMPK, Cell Signaling, 1:1000 (Gene, company, dilution)

Secondary antibody : rb, 1:10000 (Species, dilution)

Journal of Cell Science • Supplementary information

WB number	315
Development date	01.06.2022
Experiment title:	Co-IP HEK DKO AMPK +-Tg

gel A: -TG

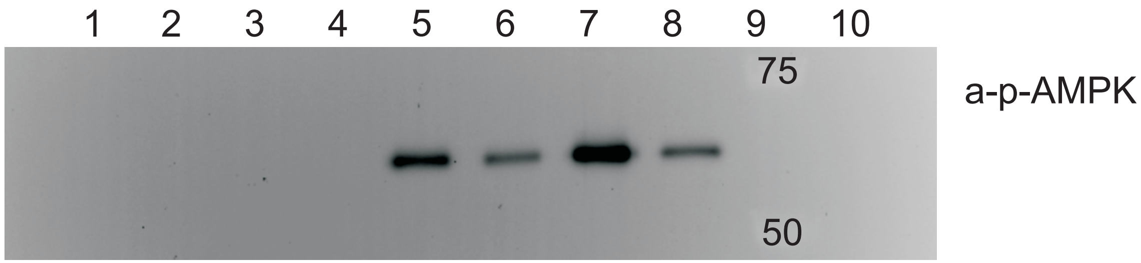

Experiment:
Transfection HEK DKO Jet Optimus 3µg DNA /6cm dish
Lysis: 30min 4°C rotating
IP: 25µL agarose, 800µg protein, over night 4°C rotating
washing 4x5min 500µL wash buffer RT, 500xg 30s
elution 25µl 50mM Tris-HCl pH 6,8 +2%SDS 5min 90°C shaking
8.5% gel, PVDF; FT and Input 25µg, entire eluate

gel B: +TG

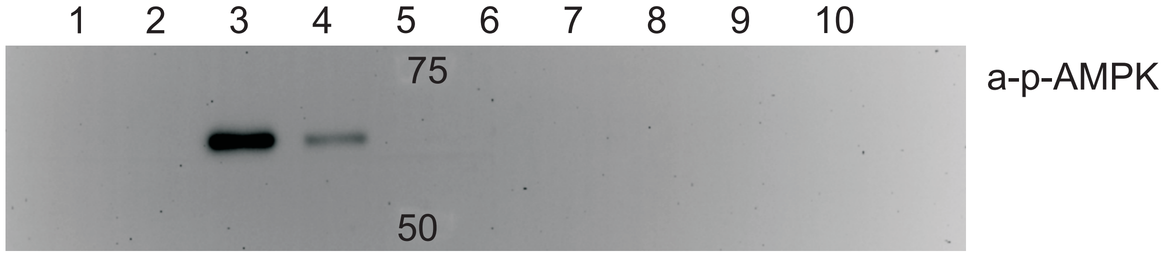

Loading scheme of gel A -TG
1. Input S2.2
2. Input S2.3
3. Input S2.2
4. Input S2.3
5. E S2.2
6. E S2.3
7. E S2.2
8. E S2.3
9. Dual color
10. FT S2.2 -Tg

Loading scheme of gel B +TG
1. Input S2.2
2. Input S2.3
3. E S2.2
4. E S2.3
5. Dual color
6. FT S2.3 -Tg
7. FT S2.2 -Tg
8. FT S2.3 -Tg
9. FT S2.2 +Tg
10. FT S2.3 +Tg

Primary Antibody: p-AMPK, Cell Signaling, 1:1000 (Gene, company, dilution)

Secondary antibody : rb, 1:10000 (Species, dilution)

Journal of Cell Science • Supplementary information

WB number	316
Development date	03./09.06.2022
Experiment title:	Co-IP HEK DKO AMPK +-Tg (315 stripped)

gel A: -TG

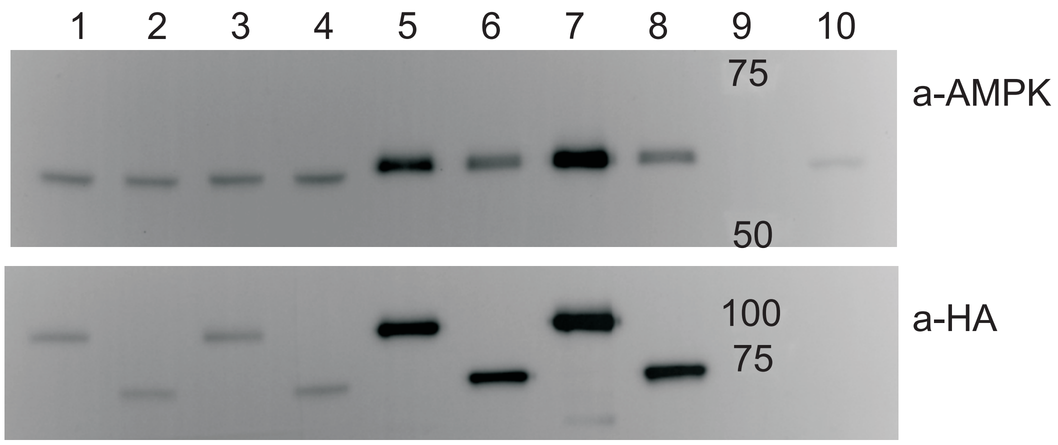

gel B: +TG

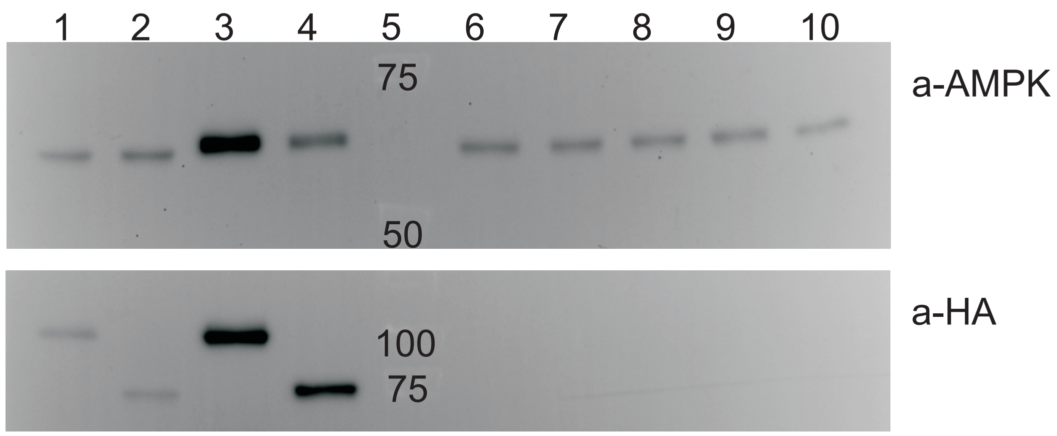

Experiment:
Transfection HEK DKO Jet Optimus 3µg DNA /6cm dish
Lysis: 30min 4°C rotating
IP: 25µL agarose, 800µg protein, over night 4°C rotating
washing 4x5min 500µL wash buffer RT, 500xg 30s
elution 25µl 50mM Tris-HCl pH 6,8 +2%SDS 5min 90°C shaking
8.5% gel, PVDF; FT and Input 25µg, entire eluate

cold stripped 20min

Loading scheme of gel A -TG
1. Input S2.2
2. Input S2.3
3. Input S2.2
4. Input S2.3
5. E S2.2
6. E S2.3
7. E S2.2
8. E S2.3
9. Dual color
10. FT S2.2 -Tg

Loading scheme of gel B +TG
1. Input S2.2
2. Input S2.3
3. E S2.2
4. E S2.3
5. Dual color
6. FT S2.3 -Tg
7. FT S2.2 -Tg
8. FT S2.3 -Tg
9. FT S2.2 +Tg
10. FT S2.3 +Tg

Primary Antibody: AMPK, Cell Signaling, 1:1000, HA3F10, Roche, 1:1000
(Gene, company, dilution)

Secondary antibody : rb, 1:10000, rat, 1:20000 (Species, dilution)

WB number	318
Development date	15./23.06.2022
Experiment title:	Co-IP HEK DKO AMPK +-Tg (2)

gel A: -TG

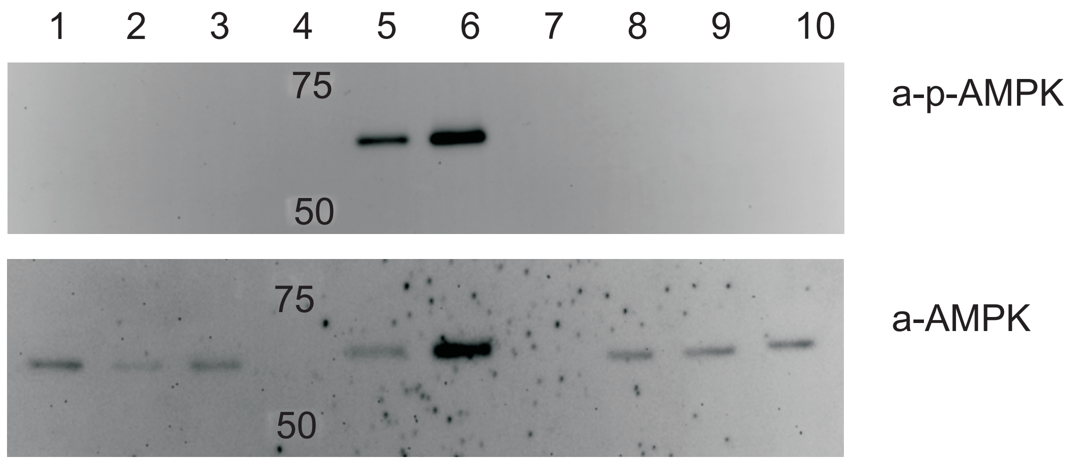

gel B: +TG

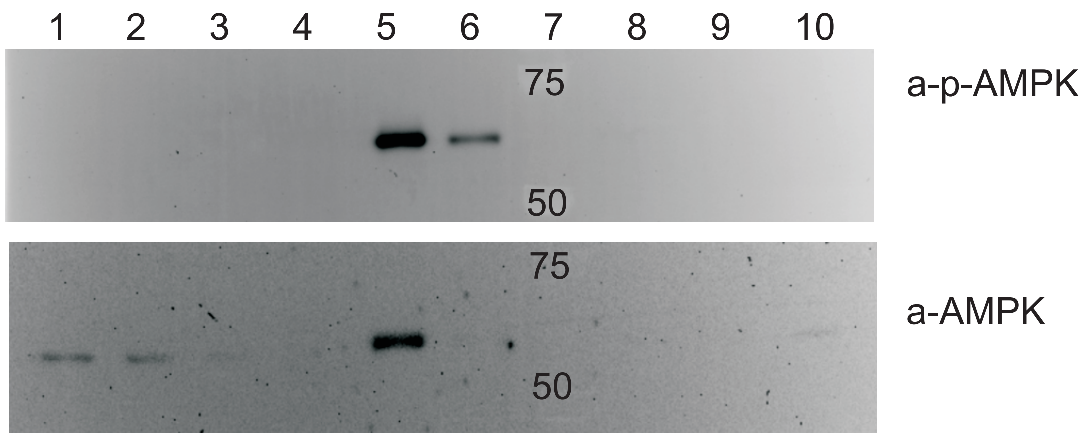

Experiment:
Transfection HEK DKO Jet Optimus 3µg DNA /6cm dish
Lysis: 30min 4°C rotating
IP: 25µL agarose, 800µg protein, over night 4°C rotating
washing 4x5min 500µL wash buffer RT, 500xg 30s
elution 25µl 50mM Tris-HCl pH 6,8 +2%SDS 5min 90°C shaking
8.5% gel, PVDF; FT and Input 25µg, entire eluate

cold stripped 20min after p-AMPK

Loading scheme of gel A -TG
1. FT ctrl
2. FT S2.2
3. FT S2.3
4. DC
5. E S2.3
6. E S2.2
7. E ctrl
8. Input S2.3
9. Input S2.2
10. Input ctrl

Loading scheme of gel B +TG
1. Input ctrl
2. Input S2.2
3. Input S2.3
4. E ctrl
5. E S2.2
6. E S2.3
7. DC
8. FT S2.2
9. FT S2.3
10. FT ctrl

Primary Antibody: p-AMPK/AMPK, Cell Signaling, 1:1000 (Gene, company, dilution)

Secondary antibody : rb, 1:10000 (Species, dilution)

WB number	324
Development date	30.06.2022
Experiment title:	Co-IP HEK DKO AMPK +-Tg (3)

gel A: -TG

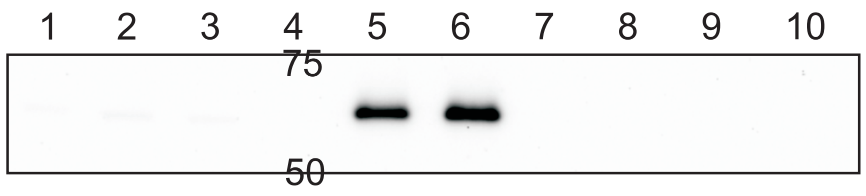

a-p-AMPK

gel B: +TG

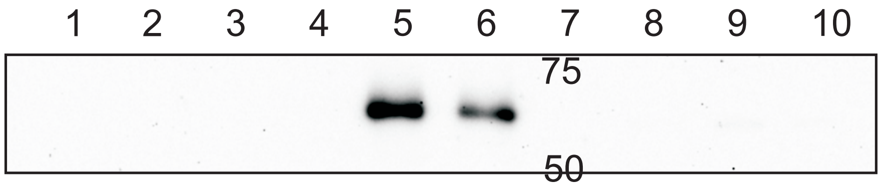

a-p-AMPK

gel C: +TG ohne ctrl

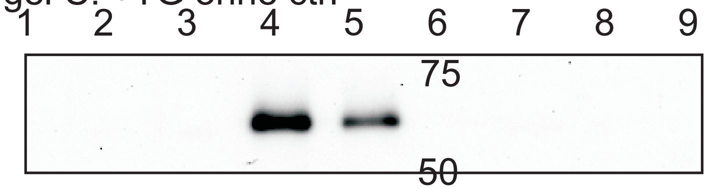

Experiment:
Transfection HEK DKO Jet Optimus 3µg DNA /6cm dish
Lysis: 30min 4°C rotating
IP: 25µL agarose, 800µg protein, over night 4°C rotating
washing 4x5min 500µL wash buffer RT, 500xg 30s
elution 25µl 50mM Tris-HCl pH 6,8 +2%SDS 5min 90°C shaking
8.5% gel, PVDF; FT and Input 25µg, entire eluate

cold stripped 20min after p-AMPK

Loading scheme of gel A -TG
1. FT ctrl
2. FT S2.2
3. FT S2.3
4. DC
5. E S2.3
6. E S2.2
7. E ctrl
8. Input S2.3
9. Input S2.2
10. Input ctrl

Loading scheme of gel B +TG
1. Input ctrl
2. Input S2.2
3. Input S2.3
4. E ctrl
5. E S2.2
6. E S2.3
7. DC
8. FT S2.2
9. FT S2.3
10. FT ctrl

Loading scheme of gel C: +TG ohne ctrl
1. DC
2. Input S2.2
3. Input S2.3
4. E S2.2
5. E S2.3
6. DC
7. FT S2.2
8. FT 2.3
9. BR
10. leer

Primary Antibody: p-AMPK, Cell Signaling, 1:1000 (Gene, company, dilution)

Secondary antibody : rb, 1:10000 (Species, dilution)

Fig. S6. Blot transparency file showing repeat experiments related to Fig. 7F-HFi

Journal of Cell Science • Supplementary information
